# Structural Insight into the Binding of TGIF1 to SIN3A PAH2 Domain through a C-Terminal Amphipathic Helix

**DOI:** 10.3390/ijms222312631

**Published:** 2021-11-23

**Authors:** Xiaoling He, Yao Nie, Heng Zhou, Rui Hu, Ying Li, Ting He, Jiang Zhu, Yunhuang Yang, Maili Liu

**Affiliations:** 1 State Key Laboratory of Magnetic Resonance and Atomic Molecular Physics, National Center for Magnetic Resonance in Wuhan, Wuhan Institute of Physics and Mathematics, Innovation Academy for Precision Measurement Science and Technology, Chinese Academy of Sciences—Wuhan National Laboratory for Optoelectronics, Wuhan 430071, China; hexl@apm.ac.cn (X.H.); nieyao2021@163.com (Y.N.); zhouheng@wipm.ac.cn (H.Z.); hurui@apm.ac.cn (R.H.); liying@apm.ac.cn (Y.L.); ht210@apm.ac.cn (T.H.); ml.liu@apm.ac.cn (M.L.); 2 University of Chinese Academy of Sciences, Beijing 100049, China

**Keywords:** transcription repressor, intrinsically disordered protein, nuclear magnetic resonance (NMR), molecular modeling, protein–protein interaction

## Abstract

TGIF1 is a transcriptional repressor playing crucial roles in human development and function and is associated with holoprosencephaly and various cancers. TGIF1-directed transcriptional repression of specific genes depends on the recruitment of corepressor SIN3A. However, to date, the exact region of TGIF1 binding to SIN3A was not clear, and the structural basis for the binding was unknown. Here, we demonstrate that TGIF1 utilizes a C-terminal domain (termed as SIN3A-interacting domain, SID) to bind with SIN3A PAH2. The TGIF1 SID adopts a disordered structure at the apo state but forms an amphipathic helix binding into the hydrophobic cleft of SIN3A PAH2 through the nonpolar side at the holo state. Residues F379, L382 and V383 of TGIF1 buried in the hydrophobic core of the complex are critical for the binding. Moreover, homodimerization of TGIF1 through the SID and key residues of F379, L382 and V383 was evidenced, which suggests a dual role of TGIF1 SID and a correlation between dimerization and SIN3A-PAH2 binding. This study provides a structural insight into the binding of TGIF1 with SIN3A, improves the knowledge of the structure–function relationship of TGIF1 and its homologs and will help in recognizing an undiscovered SIN3A-PAH2 binder and developing a peptide inhibitor for cancer treatment.

## 1. Introduction

TGIF1 (TG-interacting factor 1 or TGFβ-induced factor homeobox 1) is a transcription factor (TF) belonging to the three-amino acid loop extension (TALE) subfamily of the homeodomain (HD) protein family [1,2,3]. Through regulating the transcription of numerous genes, TGIF1 plays crucial roles in various aspects of human development and function, such as the development of fetal face and brain, hematopoiesis, bone formation and energy metabolism [4,5,6,7,8]. Gene mutations that impair TGIF1 function are associated with holoprosencephaly (HPE), a genetic disease with fetal craniofacial malformation [9,10,11,12,13]. In the last decade, increasing evidence established a close coherence of TGIF1 expression with the progression of various cancers [14,15,16,17,18,19].

**Figure 1:**
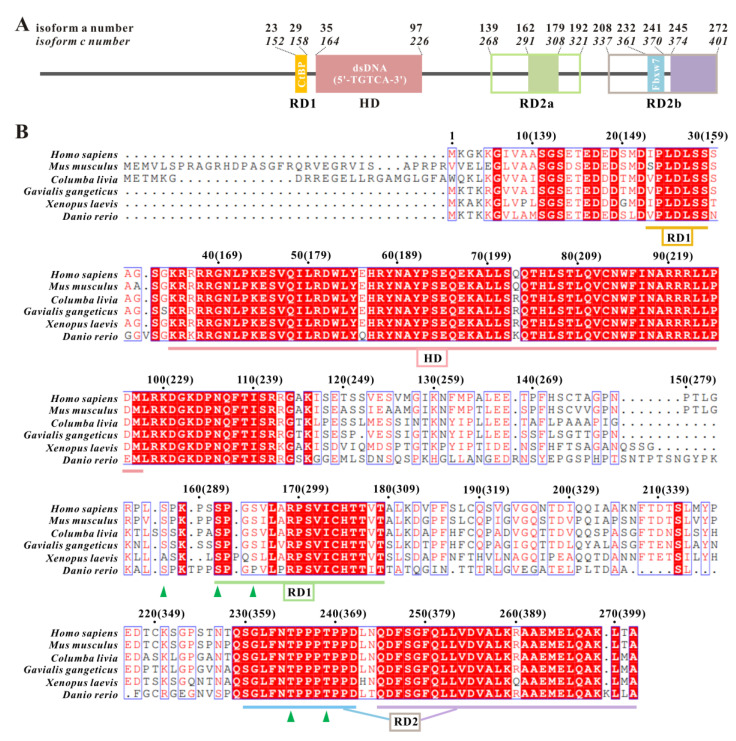
Sequence and functional domains of TGIF1. (**A**) Schematic domain structure of human TGIF1. HD, homeodomain; RD, repressive domain. The borders of respective functional domains are labeled with the residue numbers of TGIF1 isoforms a and c. The empty boxes of RD2a and RD2b represent the boundaries identified by previous functional studies, while the filled boxes within represent the boundaries of the conserved regions in sequence analyzed in this study. (**B**) Sequence alignment of TGIF1 from different vertebrates. Identical (white letters filled with red color) and similar (red letters with blue box) amino acids are denoted. The residue numbers for TGIF1 isoforms a and c (in brackets) are labeled respectively. The conserved regions in respective domains are underlined with different colors. Green arrowheads mark the phosphorylation sites.

TGIF1 functions mainly as a DNA-binding transcription repressor that interacts with general corepressors, including CtBP1/2, Sin3A and histone deacetylases (HDACs) [20,21,22]. TGIF1 was initially identified to contain 272 amino acid residues (isoform a, high-abundant form), while the following study further identified a variety of TGIF1 isoforms resulting from mRNA alternative splicing, with the longest one containing 401 residues (isoform c) [23]. Three major functional domains including HD and repressive domains 1 and 2 (RD1/2) have been demonstrated in TGIF1 (Figure 1A) [20,21,22,24]. TGIF1 HD binds to gene promoters containing a consensus sequence of 5’-TGTCA-3′, and thousands of genes have been identified to be directly regulated by TGIF1 [6,25,26,27]. Following DNA binding via the HD, TGIF1 recruits corepressor CtBP1/2 through RD1 containing a conserved “PLDLS” motif [20] and/or Sin3A and HDACs through RD2 [21,22], leading to the change of chromatin state and thereby transcription repression. Moreover, some functions of TGIF1 independent of DNA binding were also evidenced in TGFβ, nuclear receptor and Wnt signaling pathways [3,17,28,29,30].

RD2, which is located at the C-terminal of TGIF1 following the HD, contains more than 150 residues, wherein residues M256–D375 (numbered according to isoform c hereafter) are essentially disordered in structure as revealed by previous NMR studies [31,32]. Functionally, RD2 can be at least divided into two parts, previously denoted as RD2a and RD2b [24]. RD2a was suggested to mediate the interactions of TGIF1 with various proteins including Smad2 [3], PHRF1 [33], Itch [34] and Axin1/2 [17] in a cell to regulate TGFβ and Wnt signaling. RD2b harbors a phosphodegron targeted by Fbxw7 for ubiquitin-mediated degradation of TGIF1 [35]. However, the binding site of transcription corepressor SIN3A in TGIF1 RD2 remains obscure, as two independent studies, respectively, reported that RD2a and RD2b can bind to SIN3A [21,22]. Thus, the mechanism for TGIF1 to recruit SIN3A is still unknown.

Sin3A is a general transcription co-regulatory factor and commonly acts as a scaffold protein assembling the DNA-binding TFs, HDACs and other chromatin-modifying enzymes into a large complex, which facilitates transcription repression through changing the chromatin compaction [36,37]. In recent years, emerging roles of SIN3A in cancer development have been revealed [38]. Sin3A contains five defined domains including three paired amphipathic helix (PAH) domains, an HDAC-interaction domain (HID) and a C-terminal domain. Therein, PAH2 is a major docking site of diverse TFs for mediating SIN3A-targeted regulation of specific gene transcription [39,40,41,42]. Peptides and small molecule inhibitors for SIN3A PAH2 have been designed to block its binding with TFs and inhibit triple-negative breast cancer (TNBC) cell metastasis [43,44,45]. The complex structures of SIN3A PAH2 binding with Sin3A-interaction domains (SIDs) of Mad1, HBP1 and Pf1 have been solved, respectively [40,46,47]. In these structures, SIN3A PAH2 adopts a similar four-helix bundle fold with a hydrophobic cleft, into which the single-helix SID of Mad1, HBP1 or Pf1 can insert. However, as the sequence conservation of the known SIDs is low, it is hard to predict the location of the SID in other TFs, such as TGIF1, through sequence analysis.

In this study, we applied NMR spectroscopy, structure stimulation and biochemical methods to investigate the structural basis of TGIF1 for binding with SIN3A PAH2. The results reveal that the TGIF1 RD2 truncation covering residues M256–A401 was structurally disordered and adopted a monomer in solution. Therein, the region harboring residues F376–E394 contributed to the binding of SIN3A as the SID. Structure stimulation manifested that TGIF1 SID formed an amphipathic helix binding into the hydrophobic cleft of SIN3A PAH2. Furthermore, F379, L382 and V383 were identified as the key residues of TGIF1 SID for SIN3A-PAH2 binding through site-directed mutagenesis combined with yeast two-hybrid (Y2H) assay. Interestingly, although TGIF1 RD2 was not observed to dimerize in solution, Y2H assay indicated that it can dimerize via the SID in a cell, implying a potential dual role of TGIF1 SID and a relationship between dimerization and SIN3A binding of TGIF1.

## 2. Results

### 2.1. TGIF1_256–401_ Adopts an Intrinsically Disordered Structure

In order to understand the structural basis of TGIF1 for recruiting corepressor SIN3A, a recombinant TGIF1 truncation, TGIF1_256–401_, which covers RD2, was produced from *E. coli*. TGIF1_256–401_ existed in the inclusion body of bacteria lysate, different from the TGIF1_256–375_ truncation previously reported to exist in the supernatant of bacteria lysate [32], probably because more than half of the residues from F376 to A401 are hydrophobic and tend to intermolecularly aggregate. Sequence alignment among the TGIF1 homologs in different vertebrates manifested that this region is highly conserved in sequence (Figure 1B).

**Figure 2:**
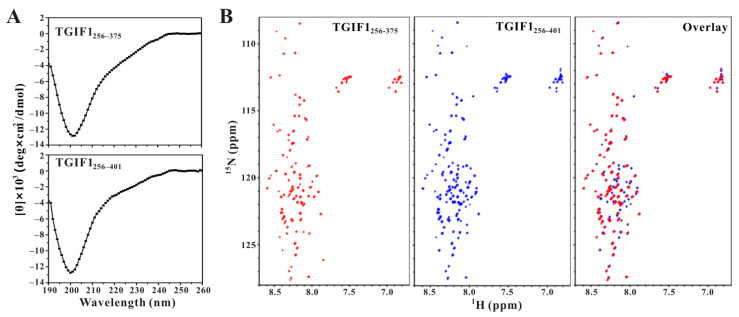
TGIF1_256–401_ adopts an intrinsically disordered structure. (**A**) CD spectra of TGIF1_256–375_ (**upper**) and TGIF1_256–401_ (**bottom**). (**B**) Comparison of ^1^H-^15^N HSQC spectra of ^15^N-labeled TGIF1_256–375_ (**left**) and TGIF1_256–401_ (**middle**) through overlaying (**right**).

The collected circular dichroism (CD) spectra of TGIF1_256–375_ and TGIF1_256–401_ similarly showed a significant negative peak near 200 nm, indicating an intrinsically disordered structure of both TGIF1 truncations (Figure 2A). NMR methods have unique advantages in studying intrinsically disordered proteins (IDPs), such as that they can provide residue-level structural information. Thus, ^15^N-labeled TGIF1_256–375_ and TGIF1_256–401_ were, respectively, prepared for the NMR study. The ^1^H–^15^N HSQC spectra of both TGIF1_256–375_ and TGIF1_256–401_ showed a narrow distribution of cross-peaks in the ^1^H dimension (concentrated between 7.7 and 8.7 ppm), consistent with the disordered structure (Figure 2B). The signal intensities of the cross-peaks were uniform, and the peak number was consistent with the residue number of each TGIF1 truncation, suggesting that they have a relatively stable conformation in solution. When the two spectra were overlaid, most cross-peaks of TGIF1_256–375_ and TGIF1_256–401_ were well overlapped except those from the additional residues F376–A401, indicating that these residues do not have significant intramolecular interaction with the residues M256–D375. Taken together, these CD and NMR data revealed an intrinsically disordered structure of TGIF1_256–401_.

### 2.2. TGIF1_376–401_ Plays a Major Role in Binding to SIN3A PAH2

**Figure 3:**
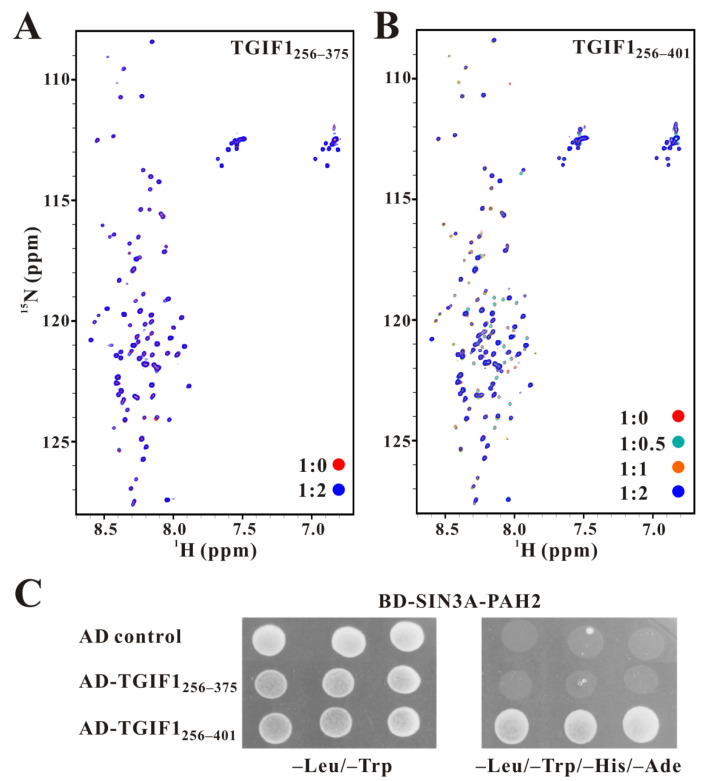
TGIF1_376–401_ interacts with SIN3A PAH2. (**A**) Superposition of ^1^H–^15^N HSQC spectra of ^15^N-labeled TGIF1_256–375_ mixed with non-labeled SIN3A PAH2 at molar ratios of 1:0 (red) and 1:2 (blue). (**B**) Superposition of ^1^H–^15^N HSQC spectra of ^15^N-labeled TGIF1_256–401_ mixed with non-labeled SIN3A PAH2 at molar ratios of 1:0 (red), 1:0.5 (cyan), 1:1 (orange) and 1:2 (blue). (**C**) Y2H experiments between SIN3A PAH2 and TGIF1 truncations. Yeast transformants harboring both AD- and BD-derived constructs were grown on SD2 (–Trp/–Leu) medium for growth control and SD4 (–Trp/–Leu/–His/–Ade) medium for the interaction test.

The binding interface of TGIF1 for SIN3A PAH2 was subsequently investigated using NMR titration of ^15^N-labeled TGIF1_256–375_ or TGIF1_256–401_ with non-labeled SIN3A PAH2. For TGIF1_256–375_, no significant change in the signal intensity and position of the cross-peaks occurred when the molar ratio of TGIF1_256–375_ vs. SIN3A PAH2 was increased from 1:0 to 1:2 (Figure 3A), suggesting that SIN3A PAH2 did not or fairly weakly interact with TGIF1_256–375_ under the condition. In contrast, when the molar ratio of TGIF1_256–401_ vs. SIN3A PAH2 was gradually increased from 1:0 to 1:2, although no obvious position migration of the cross-peaks was observed, the signal intensities of many cross-peaks significantly decreased and eventually disappeared (Figure 3B), which undoubtedly meant a substantial binding of TGIF1_256–401_ with SIN3A PAH2. Specifically, the disappeared residues were concentrated at the region covering F376 to A401, indicating that the principal binding interface of TGIF1_256–401_ for SIN3A PAH2 should be located in the C-terminal region harboring residues F376–A401.

At the same time, a Y2H experiment between SIN3A PAH2 and different TGIF1 truncations was performed, in which SIN3A PAH2 was fused with the BD domain of yeast GAL4 and TGIF1 truncations were fused with the GAL4 AD domain. The result shows that the yeast cells expressing BD-SIN3A-PAH2 and AD-TGIF1_256–401_ could grow normally on SD4 (lack of Ade, His, Leu and Trp components) medium, whereas those expressing BD-SIN3A-PAH2 and AD-TGIF1_256–375_ could not (Figure 3C), confirming the dominant role of residues F376–A401 for binding to SIN3A PAH2.

### 2.3. TGIF1_376–401_ Sequence Is Conserved among TGIF1 Homologs and Exhibits Similarity to SIDs of Other TFs

**Figure 4:**
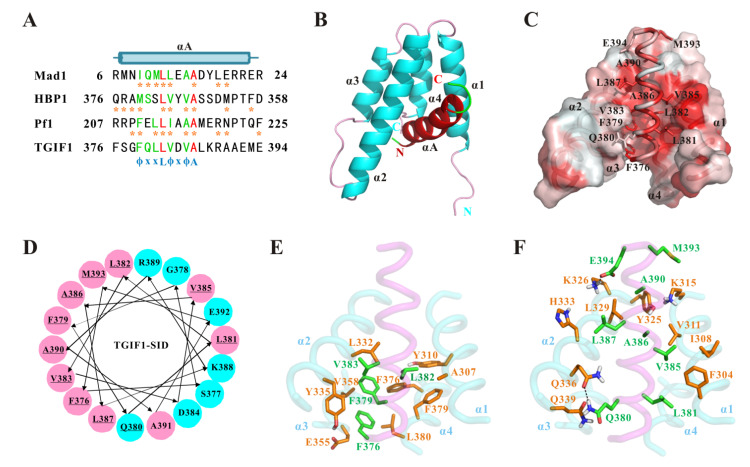
Structure model of TGIF1-SID/SIN3A-PAH2 complex. (**A**) Sequence alignment of TGIF1 SID (F376–E394) with the SIDs of Mad1, HBP1 and Pf1. Identical residues are colored in red, and similar residues are in green. The predicted secondary structure element of TGIF1 SID is shown on top, while the consensus motif is shown at bottom. The residues of Mad1, HBP1 and Pf1 SIDs showing contacts with SIN3A PAH2 in previously solved complex structures are marked with orange asterisks. (**B**) The structure model of TGIF1-SID/SIN3A-PAH2 complex is shown in cartoon representation with secondary structure elements labeled respectively. TGIF1 SID is colored in red (helix) and green (loop), while SIN3A PAH2 is colored in cyan (helix) and pink (loop). The N- and C-termini are labeled with red and cyan fonts for TGIF1 SID and SIN3A PAH2, respectively. (**C**) The structure model of TGIF1-SID/SIN3A-PAH2 complex is shown with the hydrophobic level displayed from high (red) to low (white). TGIF1 SID is shown in cartoon tube representation with the side chains of the residues interacting with SIN3A PAH2 displayed as sticks and labeled with residue number. SIN3A PAH2 is shown in surface representation with four helices labeled. (**D**) Helical wheel representation of 18 residues (F376–M393) in TGIF1 SID. Polar residues are shown in cyan circles, while nonpolar residues are in magenta circles. The underlined residues have contact with SIN3A PAH2 in the complex structure model. (**E**,**F**) are the side-chain interactions between TGIF1 SID (backbone in magenta) and SIN3A PAH2 (backbone in cyan). The side chains of TGIF1-SID residues involved in interaction are shown as sticks with carbon colored in green, and those of Sin3A PAH2 are shown as sticks with carbon colored in orange. Non-interacting parts of Sin3A PAH2 were omitted for clarity. Hydrogen bond and salt bridge are shown with black dashed lines.

As mentioned earlier, the sequences of residues F376–A401 among the TGIF1 homologs in different vertebrates are highly conserved. Considering that this region of human TGIF1 plays a major role in binding to SIN3A PAH2, the binding of SIN3A should be an evolutionally conserved function of TGIF1 for transcription regulation. Meanwhile, the sequence similarity of TGIF1 F376–A401 with the previously identified SIDs of Mad1, HBP1 and Pf1 was analyzed by sequence alignment, which manifested an eight-residue motif with moderate similarity and suggested that the residues F376–E394 were probably sufficient for SIN3A-PAH2 binding (Figure 4A). Moreover, the consensus sequence of the eight-residue motifs could be summarized as ϕ–x–x–L–ϕ–x–ϕ–A, wherein “ϕ” represents hydrophobic residue and “x” represents non-conserved residue. Inferred by the sequence similarity, TGIF1 SID may employ a similar binding model for SIN3A PAH2 as the other three SIDs. Regrettably, the cross-peaks of TGIF1 SID in complex with Sin3A PAH2 showed broadened signals and were even unable to be detected in the NMR spectrum, preventing the direct structure determination of holo TGIF1 SID and the complex.

### 2.4. Structure Model of TGIF1-SID/SIN3A-PAH2 Complex Reveals the Interaction Pattern

In order to obtain the detailed interaction mechanism, the complex structure model of TGIF1 SID and SIN3A PAH2 was established by multi-step molecular modeling (Figure 4B). In the complex structure, SIN3A PAH2 adopts a four-helix bundle structure with a deep hydrophobic pocket formed between helices α1 and α2. TGIF1 SID (F376–E394) adopts a single amphipathic α-helix with one side full of hydrophobic residues and the other side mainly containing hydrophilic residues. The helix binds to the hydrophobic pocket of SIN3A PAH2 with the hydrophobic side (Figure 4C,D). The overall complex structure is similar to the structures of SIN3A PAH2, respectively, in complex with SIDs of Mad1, HBP1 and Pf1. Interestingly, CD and NMR data suggested a major disordered structure of TGIF1 SID at the apo state, implying a potential disorder-to-order transition of TGIF1 SID upon SIN3A-PAH2 binding. This is similar to the situations of SIDs of Mad1, HBP1 and Pf1, suggesting a common behavior for PAH2 binders.

The side-chain conformations of TGIF1 SID and SIN3A PAH2 in the complex structure model were well defined, enabling a detailed analysis of interaction at residue level. The residues in the α1 and α2 helices are dominant in the pocket of SIN3A PAH2 for TGIF1-SID binding, while several residues in the α3 and α4 helices are also involved. The pocket floor is defined by the side chains of A307, Y310, L332, Y335, E355, V358, F376, F379 and L380, which conduct hydrophobic interactions with the N-terminus of TGIF1 SID including residues F376, F379, L382 and L383 (Figure 4E). The pocket edge of SIN3A PAH2 is defined by a set of residues involving F304, I308, V311, K315, Y325, K326, L329, H333, Q336 and Q339 from the α1 and α2 helices (Figure 4F). F304, I308, V311, K315, Y325, L329 and H333 make hydrophobic interactions with L381, V385, A386, L387, A390 and M393 of TGIF1. Q336 and Q339 of SIN3A make polar interactions with TGIF1 Q380 with a hydrogen bond formed between SIN3A Q336 and TGIF1 Q380. SIN3A K326 shows an electrostatic interaction with TGIF1 E394 with a salt bridge formed between their side chains. Importantly, F379, L382 and L383 of TGIF1 are deeply embedded in the pocket of SIN3A PAH2, such that they appear to be an indispensable part of the hydrophobic core of the complex. Overall, TGIF1 SID recognizes SIN3A PAH2 through an amphipathic helix in the manner of hydrophobic interaction with the nonpolar side. Generally, the interaction pattern in the structure model of the TGIF1-SID/Sin3A-PAH2 complex conferred by the conserved residues in the consensus motif “ϕ–x–x–L–ϕ–x–ϕ–A” is similar to those in the complex structures of Sin3A-PAH2/Mad1-SID, Sin3A-PAH2/HBP1-SID and Sin3A-PAH2/Pf1-SID. Nevertheless, the interaction details mediated by the non-conserved residues in TGIF1 SID vary a lot compared to those in the other three complexes.

### 2.5. F379, L382 and V383 Are Key Residues of TGIF1 SID for Binding with Sin3A PAH2

**Figure 5:**
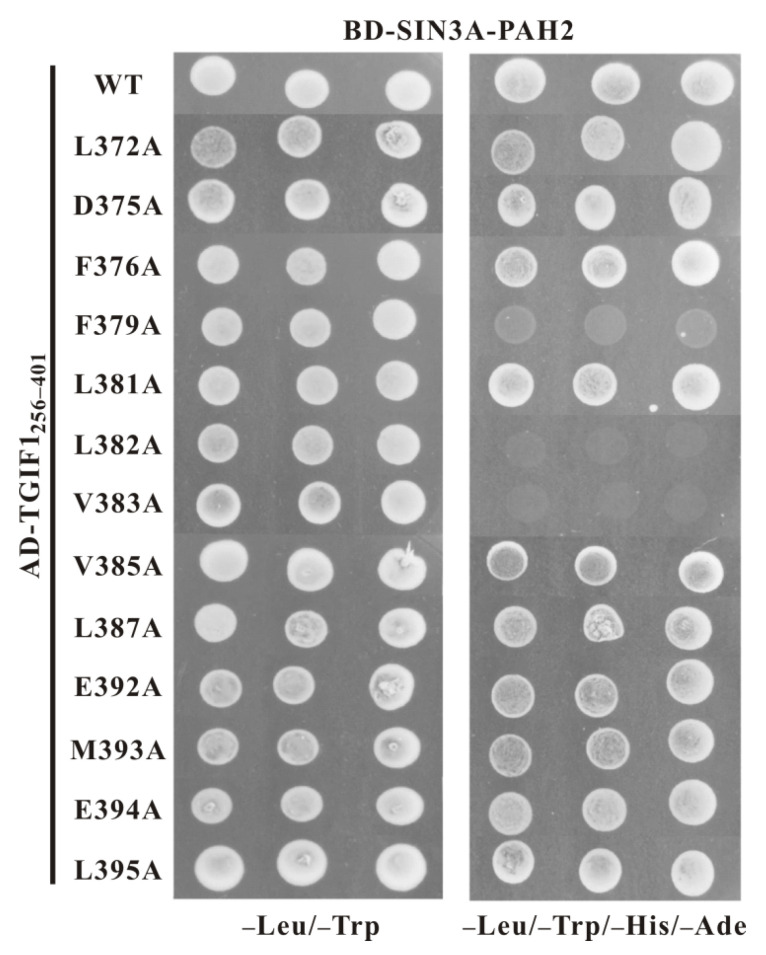
Key residues of TGIF1 SID for binding to Sin3A PAH2. Y2H experiments between SIN3A PAH2 and different versions of TGIF1_256–401_. WT, wild type. Yeast transformants harboring both AD- and BD-derived constructs were grown on SD2 (–Trp/–Leu) medium for growth control and SD4 (–Trp/–Leu/–His/–Ade) medium for the interaction test.

In order to further evaluate the importance of an individual TGIF1 residue at the binding interface for SIN3A PAH2, 13 residues of TGIF1_256–401_ with big side chains were mutated into alanine, and the resulting mutants were subsequently tested for SIN3A-PAH2 binding through a Y2H experiment. Among the 13 mutants, F379A, L382A and V383A cannot bind with SIN3A PAH2, as the yeast cells co-expressing these mutants and SIN3A PAH2 could only grow on SD2 medium but not on SD4 medium (Figure 5). Other mutants did not show obviously disrupted binding of SIN3A PAH2. These data indicate that F379, L382 and V383 are crucial for binding to SIN3A PAH2, consistent with the complex structure model, in which F379, L382 and V383 are an indispensable part of the complex hydrophobic core. Similarly, mutations of L370 and M373 of HBP1 and F210 and L213 of Pf1, which correspond to F379 and L382 of TGIF1 in sequence alignment, also impair the binding with SIN3A PAH2 [40,47], confirming a common mechanism. However, the key role of the residue at the position corresponding to TGIF1 V383 in the consensus motif was evidenced for the first time in this study, calling for more investigations of the residues at this position in SIDs of other SIN3A-PAH2 binders.

### 2.6. TGIF1 SID Mediates Homodimerization of TGIF1 in Cell

**Figure 6:**
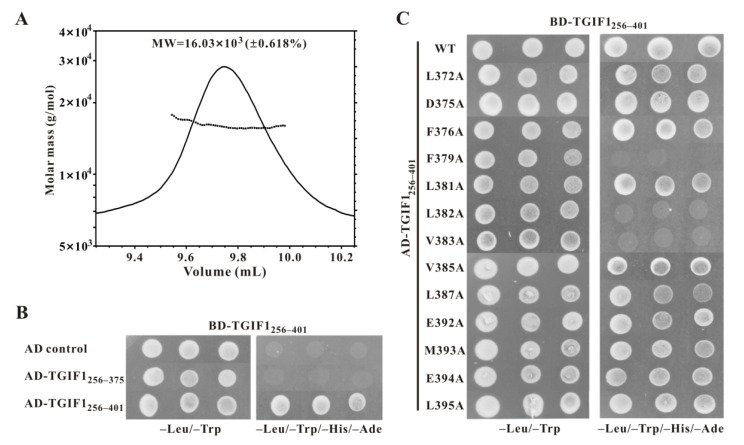
TGIF1 SID mediates homodimerization of TGIF1 in cell. (**A**) Multi-angle light scattering (MALS) assay of TGIF1_256–401_. The light scattering curve is shown in a relative scale of laser signal. The determined molar mass of individual laser signal point is shown with dot, and the average molar mass is shown as inset. (**B**) Y2H experiments of BD-TGIF1_256–401_ with AD-TGIF1_256–375_ and AD-TGIF1_256–401_ for homodimerization test. (**C**) Y2H experiments between BD-TGIF1_256–401_ and different versions of AD-TGIF1_256–401_ for homodimerization test. Yeast transformants harboring both AD- and BD-derived constructs were grown on SD2 (–Trp/–Leu) medium for growth control and SD4 (–Trp/–Leu/–His/–Ade) medium for the interaction test.

A previous study reported that the C-terminal part (residues F237–A401) of TGIF1 mediates its homodimerization in human 293 cells [48]. Thus, multi-angle light scattering (MALS) coupled with size-exclusion chromatography (SEC) was carried out for recombinant TGIF1_256–401_ to determine whether it existed as a homodimer in solution. The results show a single peak in the chromatography with a calculated average molecular weight (MW) of 16.03 × 10^3^ Da (±0.618%), which was close to the theoretical MW (15.63 kDa) of the TGIF1_256–401_ monomer (Figure 6A). This indicates that TGIF1_256–401_ exists as a monomer in solution instead of a homodimer, which may be due to the disordered structure of apo-state TGIF1_256–401_ that is insufficient for homodimer formation. At the same time, a Y2H assay was used to investigate the homodimerization of TGIF1_256–401_. The result manifested that TGIF1_256–401_ can homodimerize in a yeast cell, as the cells co-expressing AD-TGIF1_256–401_ and BD-TGIF1_256–401_ grew well on SD4 medium (Figure 6B). Furthermore, the yeast cells co-expressing AD-TGIF1_256–375_ and BD-TGIF1_256–401_ could not grow on SD4 medium (Figure 6B), indicating that the residues F376–A401 essential for binding to SIN3A PAH2 are also crucial for homodimerization of TGIF1 in a cell. The inconsistent results of MALS and Y2H may be due to the different states of TGIF1 in the two experiments.

Given that the region covering residues F376–A401 mediates the homodimerization of TGIF1 in yeast cells, the key residues for homodimerization were subsequently identified by Y2H employing the 13 AD-TGIF1_256–401_ mutants which were used in the Y2H assay with SIN3A PAH2. Among the 13 mutants, F379A, L382A and V383A could not homodimerize as the yeast cells co-expressing these mutants and BD-TGIF1_256–401_ could only grow on SD2 medium but not on SD4 medium (Figure 6C). Other mutants did not show obviously disrupted homodimerization. These data indicate that the residues F379, L382 and V383 are crucial for homodimerization, which are identical to those crucial for binding with SIN3A PAH2. Taken together, these results suggest that TGIF1-SID plays a dual role of mediating the dimerization and the binding with SIN3A.

## 3. Discussion

The homeodomain protein TGIF1 is a transcriptional repressor playing crucial roles in human development and function and is associated with HPE and various cancers. TGIF1-directed transcriptional repression of specific genes depends on the recruitment of corepressor SIN3A and subsequent HDACs to alter the chromatin accessibility, which is mediated by the previously designated RD2 of TGIF1. However, due to the inconsistent results of previous studies [21,22], to date, the exact region of TGIF1 for binding to SIN3A was not clear, and the structural basis for the binding was unknown. Here, we evidence that the residues F376–E394 of TGIF1 (designated as TGIF1 SID) play a pivotal role in binding to SIN3A PAH2 and establish a structural model for the TGIF1-SID/SIN3A-PAH2 complex. Interestingly, we reveal that TGIF1 undergoes homodimerization through the SID in a cell, implying a dual role of the SID.

### 3.1. Disorder-to-Order Transition of TGIF1 SID upon Binding to SIN3A PAH2

Previous NMR studies evidenced that the region covering residues M256–D375 of TGIF1 is disordered at the apo state in solution [31,32]. On the basis of these studies, we further evidence that the residues F376–A401 are also disordered at the apo state in this study. On the other hand, molecular modeling manifested an α-helix structure of TGIF1 F376–E394. Similarly, an α-helix conformation of TGIF1 F376–A401 was predicted in the structure model of TGIF1 by AlphaFold2 recently and was recorded with the ID of AF-Q15583-F1 in the AlphaFold protein structure database [49]. It seems that there is an inconsistency between the experiment and prediction results. However, the predicted α-helix structure of TGIF1 SID is highly possibly due to the fact that its homologous sequences with a solved helical structure are largely at the holo state, such as the SIDs of Mad1, HBP1 and Pf1, which provide the major reference information for the structure predictions by I-TASSER and AlphaFold. In fact, Mad1-SID and HBP1-SID are disordered at the apo state [40]. Thus, the predicted conformation of TGIF1 SID reasonably represents its holo state, which can be docked to SIN3A PAH2 with a high score. Similar to Mad1 SID and HBP1 SID, TGIF1 SID should undergo a disorder-to-order transition between apo and holo states. These results appeal for a cautious consideration when researchers deal with a new structure model predicted by software such as I-TASSER and AlphaFold, as proteins may adopt an experimentally undiscovered conformation, which, currently, remains not included in the reference data pool of these types of software, although they have shown remarkable success in independent assessments of accuracy.

### 3.2. Information from the Interaction Pattern of TGIF1 SID with SIN3A PAH2

Following the determination of TGIF1 F376–A401 as the major binding region of SIN3A PAH2, a complex structure model was obtained through molecular modeling in this study, which identified F376–E394 as the SID of TGIF1 and manifested a moderately conserved binding pattern between TGIF1 SID and SIN3A PAH2. Hydrophobic interactions conducted by the N-terminal F379, L382 and V383 residues of TGIF1 SID with the pocket floor of SIN3A PAH2 provide the pivotal contribution for the binding of the two proteins, as mutation of either of the three residues can abolish the binding. Sequence alignment of TGIF1 SID with SIDs of Mad1, HBP1 and Pf1 indicates that the three residues in SIDs are conserved. Moreover, previous structure studies revealed that the corresponding three residues in Mad1, HBP1 and Pf1 all make critical interactions with SIN3A PAH2, although the role of the residue corresponding to TGIF1 V383 was not subjected to mutation analysis [40,46,47]. On the other hand, F376 at the N-terminal end of TGIF1 SID and those residues interacting with the pocket edge of SIN3A PAH2 may play an auxiliary role to enhance the binding. Corresponding residues in Mad1, HBP1 and Pf1 are variable in amino acid type, indicating a specificality of the interaction pattern for an individual SIN3A binder. In general, the SIN3A-PAH2 binders have a consensus sequence of ϕ–x–x–L–ϕ–x–ϕ–A, and all form an amphipathic helix at the holo state.

TGIF1 and SIN3A are associated with many cancers. A decoy peptide containing the MAD1-SID sequence showed inhibitory activity to TNBC cells [43,45]. Identification of the TGIF1-SID sequence enriches the SID sequence pool that can be referred to when the decoy peptide targeting SIN3A is further developed in the future. Moreover, the structure model of the TGIF1-SID/SIN3A-PAH2 complex provides important knowledge for reasonable modification to increase the binding affinity of the decoy peptide. Thus, this work will help in the development of anti-cancer drugs for the clinical treatment of TGIF1-related disease.

### 3.3. Correlation between Homodimerization and SIN3A Binding of TGIF1

A previous study focusing on the relationship of TGIF1 and HPE showed that the C-terminal part of TGIF1 harboring residues F237–A401, which largely belong to the designated RD2 domain, mediates the formation of a TGIF1 homodimer using co-immunoprecipitation assay in human 293 cells [48]. The homodimerization of TGIF1 was reproduced using Y2H assay for a TGIF1_256–401_ truncation in this study, with the identifications of the exact region and key residues for homodimerization. However, recombinant TGIF1_256–401_ produced from *E. coli* existed as a monomer in solution as evidenced by the MALS-SEC assay. This draws a speculation that the homodimerization of TGIF1 in a cell is prompted by some factors not identified currently, such as post-translation modification. On the other hand, the intrinsically disordered structure of TGIF1_256–401_ may not be able to provide an effective homodimerization interface. The fact that recombinant TGIF1_256–401_ fell into the inclusion body during expression in *E. coli*, suggested that it conducted irregularly intermolecular interaction leading to insoluble aggregation. Maybe post-translation modification or environment in a eukaryotic cell can remodel the conformation of TGIF1_256–401_ to form an interface for homodimerization. Considering that homodimerization and SIN3A binding utilize the same domain and key residues, there should be an antagonism between the two behaviors of TGIF1, while which one is dominant must depend on whether it has higher binding affinity. However, it is also very likely that TGIF1_256–401_ forms dimers indirectly through binding to some adaptor protein. SIN3A may be a candidate for the adaptor protein, given that the region and key residues for TGIF1 homodimerization in a cell and binding with SIN3A are identical and there is a conserved SIN3A homolog in yeast. Currently, the role of TGIF1 homodimerization is not clear. If TGIF1 homodimerization is achieved indirectly through binding to SIN3A, it will be an additional effect of complex assembly. If TGIF1 homodimerizes directly through the SID or indirectly through binding to an adaptor protein other than SIN3A, there will be a competition between the homodimerization and SIN3A binding, which leads to a negative role in TGIF1/SIN3A-mediated transcription repression. In any event, the role of TGIF1 homodimerization needs further investigation.

### 3.4. Sequence and Function Comparison of Human TGIF Homologs

**Figure 7:**
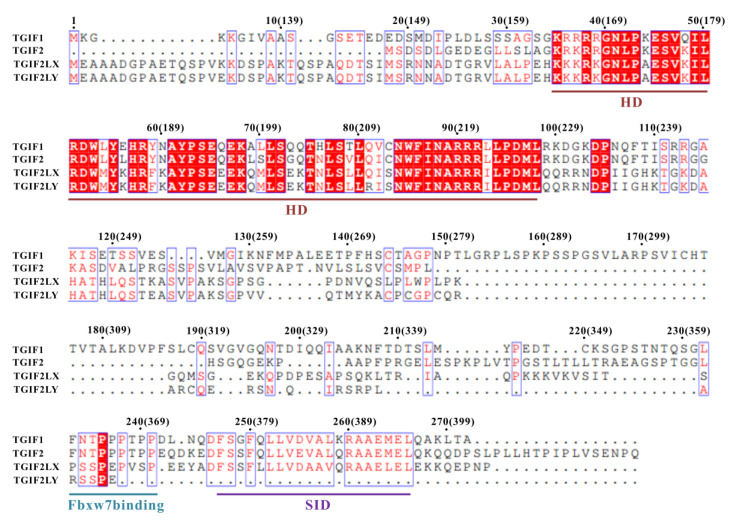
Sequence alignment of human TGIF1, TGIF2, TGIF2LX and TGIF2LY. Identical (white letters filled with red color) and similar (red letters with blue box) amino acids are denoted. The residue number for TGIF1 isoforms a and c (in brackets) are respectively labeled. The regions for homeodomain (HD), Fbxw7 binding motif and SIN3A-interacting domain (SID) in RD2b are underlined in different colors.

The human genome has four *TGIF* genes encoding TGIF1, TGIF2, TGIF2LX and TGIF2LY proteins, respectively. TGIF2 functions redundantly with TGIF1 in the transcription repression of many genes [13,27,50,51], while the functions of TGIF2LX and TGIF2LY remain obscure. Sequence alignment revealed that only one functional domain, which is the homeodomain, is unanimously included by the four TGIF proteins (Figure 7). TGIF1, TGIF2 and TGIF2LX but not TGIF2LY have the SID for binding to SIN3A PAH2, suggesting that the three proteins should utilize a similar mechanism for transcription regulation, that is, recruiting the corepressor SIN3A. Consistently with this, TGIF2 can recruit HDAC1 for transcription repression [52], which is reasonably considered to be dependent on recruiting SIN3A at first, according to current knowledge [36,37]. An Fbxw7-targeted motif can also be found in TGIF2, implying a potentially Fbxw7-mediated turnover of TGIF2 through a ubiquitin-dependent degradation pathway. However, the “PLDLS” motif for recruiting CtBP1/2 and the RD2a of TGIF1 are not contained in TGIF2, TGIF2LX and TGIF2LY, suggesting the unique molecular functions of TGIF1 among the four proteins.

## 4. Materials and Methods

### 4.1. Production of Recombinant Proteins

The coding DNA fragments of TGIF1_256–375_, TGIF1_256–401_ and SIN3A PAH2 (residues S295–N384) were cloned from human HeLa cell and inserted into a modified pET32 vector, which allows a recombinant expression of individual protein fused with a purification tag of “MHHHHHHSSGLVPRGS”. After DNA sequencing, the plasmids containing the coding sequences were respectively transformed into *E. coli* Rosetta (DE3) cell for inducing protein expression using a similar method as described previously [32].

The purification of TGIF1_256–375_ was carried out as previously described [32]. For TGIF1_256–401_ purification, following collection by centrifugation, *E. coli* cells were resuspended in solution A (20 mM Tris-HCl, 500 mM NaCl, 1 mM phenylmethylsulfonyl fluoride (PMSF), pH 7.8) for lysis by sonication. After high-speed centrifugation, the supernatant was discarded, and the pellet was washed two times with solution B (20 mM Tris-HCl, 500 mM NaCl, 2 mM EDTA, pH 7.8), followed by two times of washing with Milli-Q water. Subsequently, the pellet was solved using solution C (20 mM Tris-HCl, 500 mM NaCl, 6 M guanidine hydrochloride, pH 7.8) and clarified by high-speed centrifugation and filtration. The clarified solution was loaded onto an ÄKTAxpress™ chromatography system (GE Healthcare, Boston, MA, US) equipped with a Ni-affinity column (HisTrap IMAC HP™ column, 5 mL). The TGIF1_256–401_ protein was eluted with solution D (20 mM Tris-HCl, 500 mM NaCl, 6 M guanidine hydrochloride, 250 mM imidazole, at pH 7.8) and then dialyzed against solution E (20 mM Tris-HCl, 500 mM NaCl, pH 7.8) for several times. The purification tag was removed by thrombin through incubation at 20 °C for 3 h. The resulting TGIF1_256–401_ with only two additional residues of “GS” at its N-terminus was further purified through gel filtration chromatography, using an NGC chromatography system (Bio-Rad, Hercules, CA, US) equipped with a HiLoad 26/60 Superdex 75 column (GE Healthcare). The purified protein was concentrated to a final concentration of 0.4 mM for NMR study in solution F (90% H_2_O/10% D_2_O (*v*/*v*), 20 mM HEPES, 80 mM NaCl, 2 mM dithiothreitol (DTT), 0.05% NaN_3_, pH 6.4).

For purification of SIN3A PAH2, the harvested *E. coli* cells were resuspended in solution G (20 mM Tris-HCl, 100 mM NaCl, 1 mM PMSF, pH 8.0) for lysis by sonication. The supernatant was clarified by centrifugation and filtration and then loaded onto the ÄKTAxpress™ system equipped with a Ni-affinity column (HisTrap IMAC HP™ column, 5 mL). The protein was eluted with solution H (20 mM Tris-HCl, 100 mM NaCl, 250 mM imidazole, at pH 8.0) and dialyzed against solution I (20 mM Tris-HCl, 100 mM NaCl, pH 8.0). Subsequently, the purification tag was removed, and the resulting SIN3A PAH2 was further purified through the gel filtration chromatography mentioned above. The purified protein was exchanged into solution F and concentrated just before NMR titration.

The purities of obtained TGIF1_256–375_, TGIF1_256–401_ and SIN3A-PAH2 proteins were assessed to be over 90% by SDS-PAGE, and the MWs were verified through electrospray ionization mass spectrometry (ESI-MS).

### 4.2. Circular Dichroism

CD spectra of TGIF1_256–375_ and TGIF1_256–401_ from 190 to 260 nm were recorded on a Chirascan™ CD spectrometer (Applied Photophysics, Leatherhead, Surrey, UK) using a 0.2 cm path length quartz cell, with a step size of 1 nm and a bandwidth of 1 nm at 25 °C. Measurements were conducted with 10 μM protein in 10 mM KH_2_PO_4_, pH 6.5. Each sample was scanned three times, and the obtained spectra were averaged and subtracted with the spectrum of buffer solution (recorded as the baseline) to generate the final spectra.

### 4.3. Multi-Angle Light Scattering

Multi-angle static light scattering (MALS) analysis of TGIF1_256–401_ was carried out on a DAWN HELEOS II MALS detector (Wyatt Technology Corp., Santa Barbara, CA, USA) coupled with a Superdex^TM^ 75 10/300 GL column (GE Healthcare) at 0.5 mL/min at room temperature in a solution of 20 mM HEPES, 80 mM NaCl, 2 mM DTT, 0.05% NaN_3_, pH 6.4. The concentration of TGIF1_256–401_ was 0.2 mM. The data were analyzed using ASTRA 7.1 software package (Wyatt Technology Corp.). The weight-average molar mass was calculated according to the theoretical UV extinction coefficient (280 nm) of TGIF1_256–401_ and using a protein dn/dc value of 0.185 mL/g.

### 4.4. NMR Experiments

NMR experiments were collected on a Bruker Avance III 850 MHz spectrometer equipped with a cryogenic probe at 293 K (25 °C). The TGIF1_256–401_ concentration was 0.4 mM. The NMR data were processed using NMRPipe [53] and analyzed using Sparky [54]. NMR titration experiments were performed by mixing 0.1 mM ^15^N-labeled TGIF1_256–375_ or TGIF1_256–401_ with non-labeled SIN3A PAH2 at indicated molar ratios in solution F. After gently shaking for 1 h that allows the binding to reach equilibrium, ^1^H–^15^N HSQC spectra were collected.

### 4.5. Yeast Two-Hybrid

Yeast two-hybrid assays were performed using the Matchmaker Yeast Transformation System (Clontech, Palo Alto, CA, USA). The coding DNA fragments of TGIF1_256–375_, TGIF1_256–401_ and SIN3A-PAH2 were inserted into pGADT7 and pGBKT7 vectors, respectively. Yeast AH109 cells were co-transformed with different pairs of pGADT7 and pGBKT7 constructs as indicated and according to the manual. All yeast transformants were grown on SD2 (–Trp/–Leu) medium for transformation success test and SD4 (–Trp/–Leu/–His/–Ade) medium for prey–bait interaction test.

### 4.6. Molecular Modeling

The structure model of TGIF1 SID was built through de novo modeling using I-TASSER [55] (http://zhanglab.ccmb.med.umich.edu/I-TASSER/ (accessed on 19 November 2021)). The sequence of TGIF1 SID (F376–E394) was entered into I-TASSER as input with recommended setting for structure modeling. The first model of TGIF1 SID generated by I-TASSER and the SIN3A-PAH2 structure from PDB (ID: 2L9S.B) were used to build the complex structure model of the two proteins through molecular simulation, which included multiple steps of docking and optimization. First, initial structures for the complex were calculated using ZDOCK [56]. The model with highest score was selected from the calculated models for further optimization using the molecular docking tool ClusPro [57], which, using multiple steps, optimized the binding of the receptor and the ligand by exhaustively sampling the free energy landscape. Ten structural models that were most-populated clusters were generated, out of which the model with the highest score was refined as the final complex structure model. PyMol 2.5 and its related programs were used to analyze the structure and produce the images.

### 4.7. Sequence Alignment

Sequence alignment was carried out using Clustal Omega (http://www.clustal.org/omega/ (accessed on 19 November 2021)) and then rendered using ESPript [58] with default settings for similarity calculations. The aligned sequence IDs of TGIF1 from different vertebrates in NCBI database: *Homo sapiens*, NP_003235.1; *Mus musculus*, NP_001157547.1; *Columba livia*, XP_021139826.1; *Gavialis gangeticus*, XP_019381338.1; *Xenopus laevis*, NP_001080420.1; *Danio rerio*, NP_955861.1. The alignment result of human TGIF1 isoform c is shown because the additional residues in isoform a showed very low sequence similarity with the analyzed TGIF1 homologs from other vertebrates.

## 5. Conclusions

In conclusion, we demonstrated that TGIF1 utilizes a C-terminal motif (termed SID) ranging from F376 to E394 to bind with SIN3A PAH2. The TGIF1 SID adopts a disordered structure at the apo state, whereas it forms an amphipathic helix upon binding to SIN3A PAH2. In the complex, SIN3A PAH2 adopts a four-helix bundle structure with a deep hydrophobic cleft, into which TGIF1 SID binds through the nonpolar side of the amphipathic helix. The residues F379, L382 and V383 of TGIF1 SID buried in the hydrophobic core of the complex are critical for the binding, which are conserved residues in SIN3A-PAH2 binders. Although recombinant TGIF1_256–401_ exists as a monomer in solution, homodimerization of TGIF1 through the SID can be found in a Y2H assay, which suggests a dual role of TGIF1 SID and a correlation between homodimerization and SIN3A-PAH2 binding of TGIF1. This study provides insight into the binding mechanism of TGIF1 with SIN3A, improves the understanding of the structure–function relationship of TGIF1 and reinforces the knowledge on the sequence and structure characteristics of SIN3A-PAH2 binders. The results can be widely applied to interpret the function of TGIF1 homologs not only from human but also from other vertebrates, recognize the potential SIN3A-PAH2 binders and design a peptide inhibitor blocking SIN3A–TFs interaction for cancer treatment.

## Data Availability

Data supporting the reported results are available from the corresponding authors (Jiang Zhu and Yunhuang Yang).

## References

[1] Burglin T.R. (1997). Analysis of TALE superclass homeobox genes (MEIS, PBC, KNOX, Iroquois, TGIF) reveals a novel domain conserved between plants and animals. Nucleic Acids Res..

[2] Bertolino E., Reimund B., Wildt-Perinic D., Clerc R.G. (1995). A Novel Homeobox Protein Which Recognizes a TGT Core and Functionally Interferes with a Retinoid-responsive Motif. J. Biol. Chem..

[3] Wotton D., Lo R.S., Lee S., Massagué J. (1999). A Smad Transcriptional Corepressor. Cell.

[4] Kuang C., Xiao Y., Yang L., Chen Q., Wang Z., Conway S.J., Chen Y. (2006). Intragenic deletion of Tgif causes defectsin brain development. Hum. Mol. Genet..

[5] Horie T., Ono K., Kinoshita M., Nishi H., Nagao K., Kawamura T., Abe Y., Wada H., Shimatsu A., Kita T. (2008). TG-interacting factor is required for the differentiation of preadipocytes. J. Lipid Res..

[6] Hamid R., Brandt S.J. (2009). Transforming growth-interacting factor (TGIF) regulates proliferation and differentiation of human myeloid leukemia cells. Mol. Oncol..

[7] Saito H., Gasser A., Bolamperti S., Maeda M., Matthies L., Jähn K., Long C.L., Schlüter H., Kwiatkowski M., Saini V. (2019). TG-interacting factor 1 (Tgif1)-deficiency attenuates bone remodeling and blunts the anabolic response to parathyroid hormone. Nat. Commun..

[8] Shah A., Melhuish T.A., Fox T.E., Jr H.F.F., Wotton D. (2019). TGIF transcription factors repress acetyl CoA metabolic gene expression and promote intestinal tumor growth. Genes Dev..

[9] Gripp K.W., Wotton D., Edwards M.C., Roessler E., Ades L., Meinecke P., Richieri-Costa A., Zackai E.H., Massague J., Muenke M. (2000). Mutations in TGIF cause holoprosencephaly and link NODAL signalling to human neural axis determination. Nat. Genet..

[10] Chen C.-P., Chern S.-R., Du S.-H., Wang W. (2002). Molecular diagnosis of a novel heterozygous 268C?T (R90C) mutation inTGIF gene in a fetus with holoprosencephaly and premaxillary agenesis. Prenat. Diagn..

[11] Aguilella C., Dubourg C., Attia-Sobol J., Vigneron J., Blayau M., Pasquier L., Lazaro L., Odent S., David V. (2003). Molecular screening of the TGIF gene in holoprosencephaly: Identification of two novel mutations. Qual. Life Res..

[12] El-Jaick K., Powers S.E., Bartholin L., Myers K.R., Hahn J., Orioli I.M., Ouspenskaia M., Lacbawan F., Roessler E., Wotton D. (2007). Functional analysis of mutations in TGIF associated with holoprosencephaly. Mol. Genet. Metab..

[13] Taniguchi K., Anderson A.E., Sutherland A., Wotton D. (2012). Loss of Tgif Function Causes Holoprosencephaly by Disrupting the Shh Signaling Pathway. PLoS Genet..

[14] Yeh B.-W., Wu W.-J., Li W.-M., Li C.-C., Huang C.-N., Kang W.-Y., Liu Z.-M., Huang H.-S. (2012). Overexpression of TG-Interacting Factor Is Associated with Worse Prognosis in Upper Urinary Tract Urothelial Carcinoma. Am. J. Pathol..

[15] Willer A., Jakobsen J.S., Ohlsson E., Rapin N., Waage J., Billing M., Bullinger L., Karlsson S., Porse B.T. (2014). TGIF1 is a negative regulator of MLL-rearranged acute myeloid leukemia. Leukemia.

[16] Xiang G., Yi Y., Weiwei H.U., Weiming W. (2015). TGIF1 promoted the growth and migration of cancer cells in nonsmall cell lung cancer. Tumor Biol..

[17] Zhang M.-Z., Ferrigno O., Wang Z., Ohnishi M., Prunier C., Levy L., Razzaque M., Horne W.C., Romero D., Tzivion G. (2015). TGIF Governs a Feed-Forward Network that Empowers Wnt Signaling to Drive Mammary Tumorigenesis. Cancer Cell.

[18] Parajuli P., Singh P., Wang Z., Li L., Eragamreddi S., Ozkan S., Ferrigno O., Prunier C., Razzaque M.S., Xu K. (2019). TGIF 1 functions as a tumor suppressor in pancreatic ductal adenocarcinoma. EMBO J..

[19] Weng C.-C., Hsieh M.-J., Wu C.-C., Lin Y.-C., Shan Y.-S., Hung W.-C., Chen L.-T., Cheng K.-H. (2019). Loss of the transcriptional repressor TGIF1 results in enhanced Kras-driven development of pancreatic cancer. Mol. Cancer.

[20] Melhuish T.A., Wotton D. (2000). The Interaction of the Carboxyl Terminus-binding Protein with the Smad Corepressor TGIF Is Disrupted by a Holoprosencephaly Mutation in TGIF. J. Biol. Chem..

[21] Sharma M., Sun Z. (2001). 5?TG3? Interacting Factor Interacts with Sin3A and Represses AR-Mediated Transcription. Mol. Endocrinol..

[22] Wotton D., Knoepfler P., Laherty C.D., Eisenman R.N., Massagué J. (2001). The Smad transcriptional corepressor TGIF recruits mSin3. Cell Growth Differ. Mol. Boil. J. Am. Assoc. Cancer Res..

[23] Hamid R., Patterson J., Brandt S.J. (2008). Genomic structure, alternative splicing and expression of TG-interacting factor, in human myeloid leukemia blasts and cell lines. Biochim. Biophys. Acta (BBA)—Gene Regul. Mech..

[24] Wotton D., Lo R.S., Swaby L.-A.C., Massague J. (1999). Multiple Modes of Repression by the Smad Transcriptional Corepressor TGIF. J. Biol. Chem..

[25] Mojsin M., Popovic J., Kovacevic Grujicic N., Stevanovic M. (2012). TG-interacting factor (TGIF) downregulates SOX3 gene expression in the NT2/D1 cell line. J. Genet Genom..

[26] Pramfalk C., Melhuish T.A., Wotton D., Jiang Z.-Y., Eriksson M., Parini P. (2014). TG-interacting factor 1 acts as a transcriptional repressor of sterol O-acyltransferase. J. Lipid Res..

[27] Anderson A.E., Taniguchi K., Hao Y., Melhuish T.A., Shah A., Turner S.D., Sutherland A.E., Wotton D. (2017). Tgif1 and Tgif2 Repress Expression of the RabGAP Evi5l. Mol. Cell. Biol..

[28] Bartholin L., Powers S.E., Melhuish T.A., Lasse S., Weinstein M., Wotton D. (2006). TGIF Inhibits Retinoid Signaling. Mol. Cell. Biol..

[29] Seo S.R., Ferrand N., Faresse N., Prunier C., Abecassis L., Pessah M., Bourgeade M.F., Atfi A. (2006). Nuclear retention of the tumor suppressor cPML by the homeodomain protein TGIF restricts TGF-beta signaling. Mol. Cell.

[30] Melhuish T.A., Chung D.D., Bjerke G.A., Wotton D. (2010). Tgif1 represses apolipoprotein gene expression in liver. J. Cell. Biochem..

[31] Guca E., Sunol D., Ruiz L., Konkol A., Cordero J., Torner C., Aragon E., Martin-Malpartida P., Riera A., Macias M.J. (2018). TGIF1 homeodomain interacts with Smad MH1 domain and represses TGF-beta signaling. Nucleic Acids Res..

[32] Cai C., Nie Y., Yue X., Zhu J., Hu R., Liu M., Yang Y. (2019). Backbone and side chain resonance assignments of the C-terminal domain of human TGIF. Biomol. NMR Assign..

[33] Ettahar A., Ferrigno O., Zhang M.Z., Ohnishi M., Ferrand N., Prunier C., Levy L., Bourgeade M.F., Bieche I., Romero D.G. (2013). Identification of PHRF1 as a tumor suppressor that promotes the TGF-beta cytostatic program through selective release of TGIF-driven PML inactivation. Cell Rep..

[34] Demange C., Ferrand N., Prunier C., Bourgeade M.-F., Atfi A. (2009). A Model of Partnership Co-Opted by the Homeodomain Protein TGIF and the Itch/AIP4 Ubiquitin Ligase for Effective Execution of TNF-α Cytotoxicity. Mol. Cell.

[35] Bengoechea-Alonso M.T., Ericsson J. (2010). Tumor suppressor Fbxw7 regulates TGFbeta signaling by targeting TGIF1 for degradation. Oncogene.

[36] Kadamb R., Mittal S., Bansal N., Batra H., Saluja D. (2013). Sin3: Insight into its transcription regulatory functions. Eur. J. Cell Biol..

[37] Adams G.E., Chandru A., Cowley S.M. (2018). Co-repressor, co-activator and general transcription factor: The many faces of the Sin3 histone deacetylase (HDAC) complex. Biochem. J..

[38] Bansal N., David G., Farias E., Waxman S. (2016). Emerging Roles of Epigenetic Regulator Sin3 in Cancer. Adv. Cancer Res..

[39] Eilers A.L., Billin A., Liu J., Ayer D. (1999). A 13-Amino Acid Amphipathic α-Helix Is Required for the Functional Interaction between the Transcriptional Repressor Mad1 and mSin3A. J. Biol. Chem..

[40] Swanson K.A., Knoepfler P.S., Huang K., Kang R.S., Cowley S.M., Laherty C.D., Eisenman R.N., Radhakrishnan I. (2004). HBP1 and Mad1 repressors bind the Sin3 corepressor PAH2 domain with opposite helical orientations. Nat. Struct. Mol. Biol..

[41] Shi X., Garry D.J. (2012). Sin3 interacts with Foxk1 and regulates myogenic progenitors. Mol. Cell. Biochem..

[42] Zhang J.-S., Moncrieffe M.C., Kaczynski J., Ellenrieder V., Prendergast F.G., Urrutia R., Haracska L., Johnson R.E., Unk I., Phillips B. (2001). A Conserved α-Helical Motif Mediates the Interaction of Sp1-Like Transcriptional Repressors with the Corepressor mSin3A. Mol. Cell. Biol..

[43] Farias E.F., Petrie K., Leibovitch B., Murtagh J., Chornet M.B., Schenk T., Zelent A., Waxman S. (2010). Interference with Sin3 function induces epigenetic reprogramming and differentiation in breast cancer cells. Proc. Natl. Acad. Sci. USA.

[44] Kwon Y.-J., Petrie K., Leibovitch B., Zeng L., Mezei M., Howell L., Gil V., Christova R., Bansal N., Yang S. (2015). Selective Inhibition of SIN3 Corepressor with Avermectins as a Novel Therapeutic Strategy in Triple-Negative Breast Cancer. Mol. Cancer Ther..

[45] Kwon Y.-J., Leibovitch B.A., Bansal N., Pereira L., Chung C.-Y., Ariztia E.V., Zelent A., Farias E.F., Waxman S. (2016). Targeted interference of SIN3A-TGIF1 function by SID decoy treatment inhibits Wnt signaling and invasion in triple negative breast cancer cells. Oncotarget.

[46] Brubaker K., Cowley S.M., Huang K., Loo L., Yochum G.S., Ayer D.E., Eisenman R.N., Radhakrishnan I. (2000). Solution Structure of the Interacting Domains of the Mad–Sin3 Complex: Implications for Recruitment of a Chromatin-Modifying Complex. Cell.

[47] Kumar G.S., Xie T., Zhang Y., Radhakrishnan I. (2011). Solution structure of the mSin3A PAH2-Pf1 SID1 complex: A Mad1/Mxd1-like interaction disrupted by MRG15 in the Rpd3S/Sin3S complex. J. Mol. Biol..

[48] Ferrand N., Demange C., Prunier C., Seo S.R., Atfi A. (2006). A mechanism for mutational inactivation of the homeodomain protein TGIF in holoprosencephaly. FASEB J..

[49] Jumper J., Evans R., Pritzel A., Green T., Figurnov M., Ronneberger O., Tunyasuvunakool K., Bates R., Žídek A., Potapenko A. (2021). Highly accurate protein structure prediction with AlphaFold. Nature.

[50] Powers S.E., Taniguchi K., Yen W., Melhuish T.A., Shen J., Walsh C.A., Sutherland A.E., Wotton D. (2010). Tgif1 and Tgif2 regulate Nodal signaling and are required for gastrulation. Development.

[51] Melhuish T.A., Taniguchi K., Wotton D. (2016). Tgif1 and Tgif2 Regulate Axial Patterning in Mouse. PLoS ONE.

[52] Melhuish T.A., Gallo C.M., Wotton D. (2001). TGIF2 Interacts with Histone Deacetylase 1 and Represses Transcription. J. Biol. Chem..

[53] Delaglio F., Grzesiek S., Vuister G.W., Zhu G., Pfeifer J., Bax A. (1995). NMRPipe: A multidimensional spectral processing system based on UNIX pipes. J. Biomol. NMR.

[54] Goddard T.D., Kneller D.G. (2000). SPARKY 3.

[55] Yang J., Zhang Y. (2015). I-TASSER server: New development for protein structure and function predictions. Nucleic Acids Res..

[56] Pierce B.G., Wiehe K., Hwang H., Kim B.-H., Vreven T., Weng Z. (2014). ZDOCK server: Interactive docking prediction of protein-protein complexes and symmetric multimers. Bioinformatics.

[57] Kozakov D., Hall D.R., Xia B., Porter K.A., Padhorny D., Yueh C., Beglov D., Vajda S. (2017). The ClusPro web server for protein–protein docking. Nat. Protoc..

[58] Robert X., Gouet P. (2014). Deciphering key features in protein structures with the new ENDscript server. Nucleic Acids Res..

